# Macro- and microelements in serum and seminal plasma as biomarkers for bull sperm cryotolerance

**DOI:** 10.1186/s13028-021-00590-2

**Published:** 2021-07-05

**Authors:** Maja Zakošek Pipan, Petra Zrimšek, Breda Jakovac Strajn, Katarina Pavšič Vrtač, Tanja Knific, Janko Mrkun

**Affiliations:** 1 Clinic for Reproduction and Large Animals, Veterinary Faculty, University of Ljubljana, Gerbičeva 60, 1000 Ljubljana, Slovenia; 2 Institute of Preclinical Sciences, Veterinary Faculty, University of Ljubljana, Gerbičeva 60, 1000 Ljubljana, Slovenia; 3 Institute of Food Safety, Feed and Environment, Veterinary Faculty, University of Ljubljana, Gerbičeva 60, 1000 Ljubljana, Slovenia

**Keywords:** Blood serum, Bull semen, Cryopreservation, Microelements, Quality, Seminal plasma

## Abstract

**Background:**

Wide variation in fertility rates is observed when using frozen bull semen, even when the bulls have met quality standards for semen production. Therefore, a simple and reliable test to assess the freezing potential of bull semen based on the analysis of fresh semen or blood would be of great value. Attention is now turning to assessment of seminal plasma components such as proteins and elements. In the present study, the concentrations of macro- and microelements in fresh bull semen plasma and in serum and their correlation with quality characteristics of fresh semen and with semen quality after freezing and thawing were determined. Ejaculates were collected from 30 mature bulls, and semen volume, concentration, sperm motility, morphology, tail membrane integrity, plasma membrane permeability and DNA fragmentation were determined on the day of collection and after freezing and thawing. The concentrations of macroelements (Na, Mg, K and Ca) and microelements (Cu, Fe, Zn and Se) were determined in the seminal plasma and serum. The semen samples were classified into satisfactory and unsatisfactory groups according to the fresh semen quality.

**Results:**

Zinc and Se levels measured in serum were associated with almost all fresh and frozen-thawed semen quality characteristics, while Fe levels were associated only with acrosomal defects in fresh semen. Zinc and Fe levels in fresh seminal plasma were associated with various quality characteristics of fresh and frozen-thawed semen, while Se level in fresh seminal plasma was not associated with any of the semen quality characteristics.

**Conclusions:**

Microelements were shown to be useful as biomarkers involved in the analysis of bull sperm quality and could be used as an additional tool to predict bull semen quality after freezing and thawing. Our results confirm that the analysis of Zn and Se levels in serum and Zn, Cu and Fe levels in fresh seminal plasma can provide information to discriminate between bull semen samples with spermatozoa with high or low cryotolerance.

## Background

Sperm quality evaluation is of economic importance in breeding management because it allows the selection of bulls with good reproductive performance. Bulls are evaluated for health, libido and mating ability, and their semen is evaluated for volume, sperm concentration, motility, viability, morphology and the ability to withstand freezing and thawing processes [1, 2]. Semen used for artificial insemination (AI) of cows should be of high quality. However, conventional estimates of fresh semen quality are not sensitive enough to distinguish between samples that differ in predictive quality after storage [2]. Moreover, the fertility of frozen bull semen varies widely, even if the bulls have met the quality standards for semen production [3]. The most accurate method for testing bull fertility is in vivo insemination of many fertile females, but this method is time consuming, expensive for routine use, and allows only a limited number of bulls to be tested at any given time [4]. Therefore, developing a simple, inexpensive, accurate, precise, and reliable test to assess the freezing potential of bull semen based on the analysis of fresh semen or blood would be of great value. Attention is now turning to the evaluation of other components of semen, such as fertility-associated proteins [5, 6] and macro- and microelements [7–9]. Elements are known to play an important role in bovine health and reproduction [10], and several studies have shown that element imbalance affects the reproductive performance of bulls and that elements such as calcium (Ca), copper (Cu), iron (Fe), magnesium (Mg), selenium (Se) and zinc (Zn) are essential for reproduction and that their deficiency can lead to degenerative changes in spermatogenesis [7, 11–13]. In humans, routine seminal plasma assessment includes the analysis of some microelements, such as Zn and Se, which are associated with sperm quality because of their antioxidant properties [14, 15].

Recently, enrichment of a sperm extender with Se nanoparticles at a concentration of 1.0 μg/mL was shown to improve bull sperm quality after thawing and consequently increase in vivo fertility rates by reducing apoptosis, lipid peroxidation and cryopreservation-induced sperm damage [7]. Moreover, our previous study in boars showed that microelements are involved in boar sperm quality and could serve as an additional tool to predict sperm quality after storage [16].

The aim of the present study was to determine the concentrations of macro- and microelements in fresh bull seminal plasma and serum and assess the relationships of these factors with sperm quality characteristics in fresh semen and in semen after freezing and thawing.

## Methods

### Experimental animals

Semen ejaculates were obtained from 30 mature (2–3 years old) and healthy breeding bulls kept in a local AI center (Agriculture and Forestry Institute Ptuj, Slovenia). We obtained one ejaculate per bull, and all bulls underwent a regular health check immediately prior to semen collection. Only bulls that were healthy were selected for the study. Semen collection was performed in October and November. All bulls included in the experiment were Simmental cattle, and they were in a regular semen collection program for insemination purposes and kept under standard conditions regarding feeding and management. According to Slovenian legislation (National Assembly of the Republic of Slovenia, 2013) covered by Directive 2010/63/EU (The European Parliament and the Council, 2010), ethical approval was not required for the experiments because only noninvasive procedures were used in this study.

### Blood collection

According to Slovenian veterinary regulations, all breeding bulls must be examined annually for various diseases. Therefore, as part of the regular examination, blood was taken from the bulls by venipuncture with a vacutainer needle and 10 mL plain red top tubes. Two red top tubes were collected from each bull, and approximately 16 ml blood was collected. The blood samples for microelement analysis were left at room temperature for 30 min and then centrifuged at 1500 g at 4 °C for 15 min. No visible hemolysis was observed in the samples taken. The blood serum was then immediately separated, aliquoted and stored at − 80 °C until the macro- and microelement concentrations were assayed.

### Collection of semen and seminal plasma preparation

Semen samples were obtained from adult breeding bulls on a regular collection schedule using a teaser bull and an artificial vagina. After collection of the samples, 1 mL of the semen was used for initial semen evaluation (motility and sperm concentration). For the initial analysis, motility was determined subjectively under an optical phase microscope (Olympus CX31/41) and the concentration was determined using a photometer (Photometer SDM 5, Minitüb, Germany). Based on the initial sperm concentration, the raw ejaculate was diluted in BullXcell extender (IMV Technologies, France) containing bidistilled water, TRIS, glycerol, citric acid, sugars, buffers, antibiotics, and fresh egg yolk at a ratio of 1:4 to achieve a concentration of 15  ×  10^6^ total sperm per 0.25 mL insemination dose. Semen straws (IMV Technologies, France) were filled according to routine procedures using an IS4 machine (IMV Technologies, France), placed in an insulated box (to slow the temperature drop) and stored in a refrigerator at 4 °C. Straws from each ejaculate were frozen 6 h after dilution and then stored in liquid nitrogen at − 196 °C. Samples were evaluated after thawing at 37 °C for 30 s in a water bath, and they were thawed at least 1 week after freezing and analyzed for post-thaw characteristics.

Semen samples were centrifuged at 800*g* for 10 min at room temperature. The supernatants were removed and centrifuged again at 13,000*g* for 15 min at 4 °C to separate seminal plasma [16]. Seminal plasma was then aliquoted and frozen at − 80 °C until assayed for macro- (Na, K, Mg, Ca) and microelements (Fe, Cu, Zn and Se).

### Sample preparation and basic semen characteristics

Motility and progressive motility in fresh and frozen semen samples were determined with a computer-assisted motility analyzer (AndroVision, Minitüb, Germany) using an optical phase microscope (Olympus CX31/41 and BX series) with a Leja counting chamber (Minitüb, Germany). The software was set using the manufacturer’s recommendation, with a slight adjustment to allow for clear identification of all spermatozoa. Settings were configured as follows: 10 fields, region of particle control 10–18 μm, depth of field 20 μm and temperature of the microscope plate 37–37.5 °C. Spermatozoa with (amplitude of lateral head) ALH  <  1.5 and straight‐line velocity (VSL)  <  12 were considered immotile (otherwise considered motile), spermatozoa with VSL  <  48 and with a curvilinear velocity (VCL)  <  60 were considered locally motile (otherwise considered progressively motile), spermatozoa with a radius  >  9 and radius  <  90 and rotation  >  0.7 were considered to have circular motility, and spermatozoa with a VCL  <  90 were considered slow motile (otherwise considered fast motile). A 10 μl aliquot of the prewarmed semen sample (5 min at 37 °C) was placed on a prewarmed Leja chamber (37–37.5 °C), and pictures of ten fields were taken (100  ×). For the reliability of the analysis, each measurement was performed twice.

Concentrations of raw and diluted semen before freezing were measured using a photometer (Photometer SDM 5, Minitüb, Germany) according to Indriastuti et al. [17].

The morphology and viability of 200 spermatozoa in fresh and frozen semen samples were assessed using eosin-nigrosin staining (Morphology Stain, Society for Theriogenology). Acrosome defects were also assessed using the oil immersion objective of a light microscope. A total of 200 sperm cells per slide were counted to distinguish between normal acrosome and acrosome abnormalities, such as absence of the acrosome, swelling or thickening, especially at the anterior tip of the sperm cell, and irregularities in the shape of the acrosome.

Samples were divided into two groups according to the quality characteristics of fresh semen based on the rules on conditions for the breeding of domesticated animals (Official Gazette of the Republic of Slovenia, No. 51/07 and 35/15; EVA 2001-2311-0094):

1. Satisfactory group (group SAT; N  =  15), which met all the requirements for satisfactory semen: motility  ≥  75%, progressive motility  ≥  60%, morphologically normal spermatozoa  ≥  75%.

2. Unsatisfactory group (UNSAT group; N  =  15), which did not meet at least one of the above requirements for satisfactory semen characteristics.

### Specific semen characteristics

#### Hypoosmotic swelling test (HOST)

A HOST was used to assess the integrity of the caudal membrane as a test of sperm function. Ten microliters of semen sample were gently mixed with 100 μL of hypoosmotic solution (150 mOsm/kg sodium citrate  ×  2H_2_O and fructose), both at 37 °C. After incubation at 37 °C in warm water for one hour, 200 spermatozoa per sample were examined under a light microscope at a magnification of  ×  400. Spermatozoa were classified as HOST positive if they showed signs of swelling (swollen head and coiled tails).

### DNA fragmentation

DNA integrity was assessed using a commercial assay (Sperm Bos-Halomax; Halotech DNA SL, Spain) based on a sperm dispersion test specifically designed for bull semen. Semen samples were processed according to the manufacturer’s instructions. Briefly, to a vial containing 50 µL of liquid low melting point agarose, 25 µL of the semen sample was added at 37 °C. The pretreated slides provided with the commercial assay were placed on a chilled glass plate at 4 °C. Two microliters of the cell suspension were applied to the treated area of the chilled slide and covered with a coverslip for 5 min. The coverslip was then carefully removed, and the slides were placed in 10 mL of the lysing solution at room temperature for 5 min. Finally, the slides were washed in distilled water for 5 min and dehydrated in sequential ethanol solutions for 2 min each (70, 90, and 100%). After drying, the samples were stained with a commercial fluorescence microscopy green staining kit (Halotech DNA, Spain) according to the instructions. Sperm chromatin dispersion was assessed using a fluorescence filter (Olympus U-MNIBA3; excitation at 497 nm and emission at 520 nm) at 400  ×  magnification (Olympus BX40). At least 300 spermatozoa were counted per semen sample, and samples were examined in duplicate.

The spermatozoa were divided into two groups according to the form of the halo effect. Group 1 contained spermatozoa with normal halo effects, i.e., intact DNA in which there was a clearly visible halo around the head, such as the diameter of the nucleus, and Group 2 contained spermatozoa with small, absent, or large halo effects.

### Analysis of macro- and microelements

Microwave digestion of the samples was performed using a MARS 5 Microwave Acceleration Reaction System (CEM, Matthews, NC). Seminal plasma samples (0.4–1 mL) and blood serum samples (1 mL) were transferred into a 100 mL Teflon vessel, and 3 mL 65% nitric acid, 0.5 mL 30% hydrogen peroxide and 4.5 mL Milli-Q water were added. The samples were digested in a closed 12 vessel microwave system at 200 °C for 30 min. After cooling to room temperature, the solutions were diluted with Milli-Q water, and the concentrations of elements were determined by inductively coupled plasma mass spectrometry (Varian 820-MS, Mulgrave, Australia). Argon was used as the carrier gas, and the isotopes ^57^Fe, ^63^Cu, ^66^Zn, and ^78^Se were selected as analytical masses in inductively coupled plasma mass spectrometry (ICP-MS) normal sensitivity mode. A collision reaction interface (CRI) was used for measurements of Se to reduce common polyatomic interferences.

### Statistical analysis

Statistical analysis was performed using R statistical software, version 3.5.2 (R Foundation for Statistical Computing, Vienna, Austria, 2018). A P value  <  0.05 was considered as significant. The results of semen characteristics and micro- and macroelements in seminal plasma and blood serum are presented with basic descriptive statistics.

Statistical comparison of the results obtained on fresh semen and after freezing thawing for each semen characteristic, macro- and microelement in SAT and UNSAT groups as well as between macro- and microelement in blood serum and seminal plasma was performed with Wilcoxon rank sum test, since the data were not normally distributed in all groups. Correlations between macro- and microelements in blood serum and seminal plasma were assessed with Spearman’s rank correlation coefficients, and Holm adjusted P values were used to determine statistical significance.

Multivariate linear regression analysis was used to test whether the concentrations of Fe, Se and Zn in blood serum and seminal plasma significantly predicted the characteristics of fresh and frozen-thawed semen. These elements were selected based on significant correlations with semen quality characteristics. To better meet the normality assumption, we used a log transformation of the variable acrosome defects and a cubic transformation of the variable’s motility, normal tail membrane integrity, normal morphology, progressive motility, and viability. Multicollinearity was tested with variance inflation factors and values  <  5 were considered below the critical level. The normality assumption of the models was evaluated using the quantile–quantile plots of the residuals.

## Results

### Semen quality characteristics of bull semen samples in the satisfactory and unsatisfactory groups

The median concentration of spermatozoa in the semen ejaculates was 1.48  ×  10^9^ sperm/mL, with a lower quartile of 1.03  ×  10^9^ sperm/mL and an upper quartile of 2.02  ×  10^9^ sperm/mL. Characteristics of semen quality in satisfactory (SAT) (N  =  15) and unsatisfactory (UNSAT) (N  =  15) groups for fresh semen samples and for semen samples after freezing and thawing are included in Table 1.

**Table 1:** Semen quality characteristics of bull semen samples for the satisfactory group and unsatisfactory group

Semen characteristics (%)	Fresh semen					
	Satisfactory group (N = 15)^a^			Unsatisfactory group (N = 15)^a^		
	Median	Lower quartile	Upper quartile	Median	Lower quartile	Upper quartile
Motility	85.00	85.00	90.00	60.00	50.00	60.00
Progressive motility	75.00	70.00	77.50	40.00	30.00	52.50
Viability	91.50	90.25	94.13	76.25	67.38	85.38
Normal morphology	86.00	83.00	89.00	68.50	50.75	77.50
Acrosome defects	2.50	1.25	3.25	5.00	3.25	11.75
Normal tail membrane integrity	93.00	91.50	96.00	82.50	74.00	87.50
DNA fragmentation	5.83	3.58	7.25	9.83	7.00	13.25

Semen characteristics (%)	Frozen-thawed semen					
	Satisfactory group (N = 15)			Unsatisfactory group (N = 15)		
	Median	Lower quartile	Upper quartile	Median	Lower quartile	Upper quartile
Motility	80.00	74.00	85.00	53.00	48.25	68.25
Progressive motility	60.00	57.13	64.75	32.50	20.63	51.50
Viability	89.25	85.13	90.88	64.00	58.38	82.75
Normal morphology	81.00	74.75	84.00	60.50	41.50	72.00
Acrosome defects	6.50	5.75	9.75	13.00	8.75	17.25
Normal tail membrane integrity	90.50	86.75	93.50	73.50	67.75	79.75
DNA fragmentation	8.50	5.42	9.25	15.67	11.75	20.6

^a^For all measured semen characteristics, statistically significant differences between satisfactory and unsatisfactory groups were observed (P  <  0.05) in fresh and frozen-thawed semen

### Concentration of elements measured in fresh seminal plasma and in blood serum

Concentrations of elements were measured on the day of collection in seminal plasma and in blood serum and are presented in Table 2.

**Table 2:** Concentration of elements measured in seminal plasma and in blood serum on the day of collection

Elements (mg/L)	Seminal plasma			Serum		
	Median	Lower quartile	Upper quartile	Median	Lower quartile	Upper quartile
Na	3387.82	2842.02	3429.06	3387.82	3101.11	3434.56
Mg	25.72	23.64	61.32	22.07	20.28	24.34
K	231.75	192.87	1081.99	187.51	161.36	199.91
Ca	103.78	96.38	255.10	93.09	81.05	97.45
Fe	2.66	2.19	2.88	2.26	2.05	2.71
Cu	0.87	0.33	1.05	0.90	0.77	1.05
Zn	1.10	0.96	3.48	3.53	3.09	4.12
Se	0.09	0.07	0.31	0.49	0.43	0.61

**Fig. 1:**
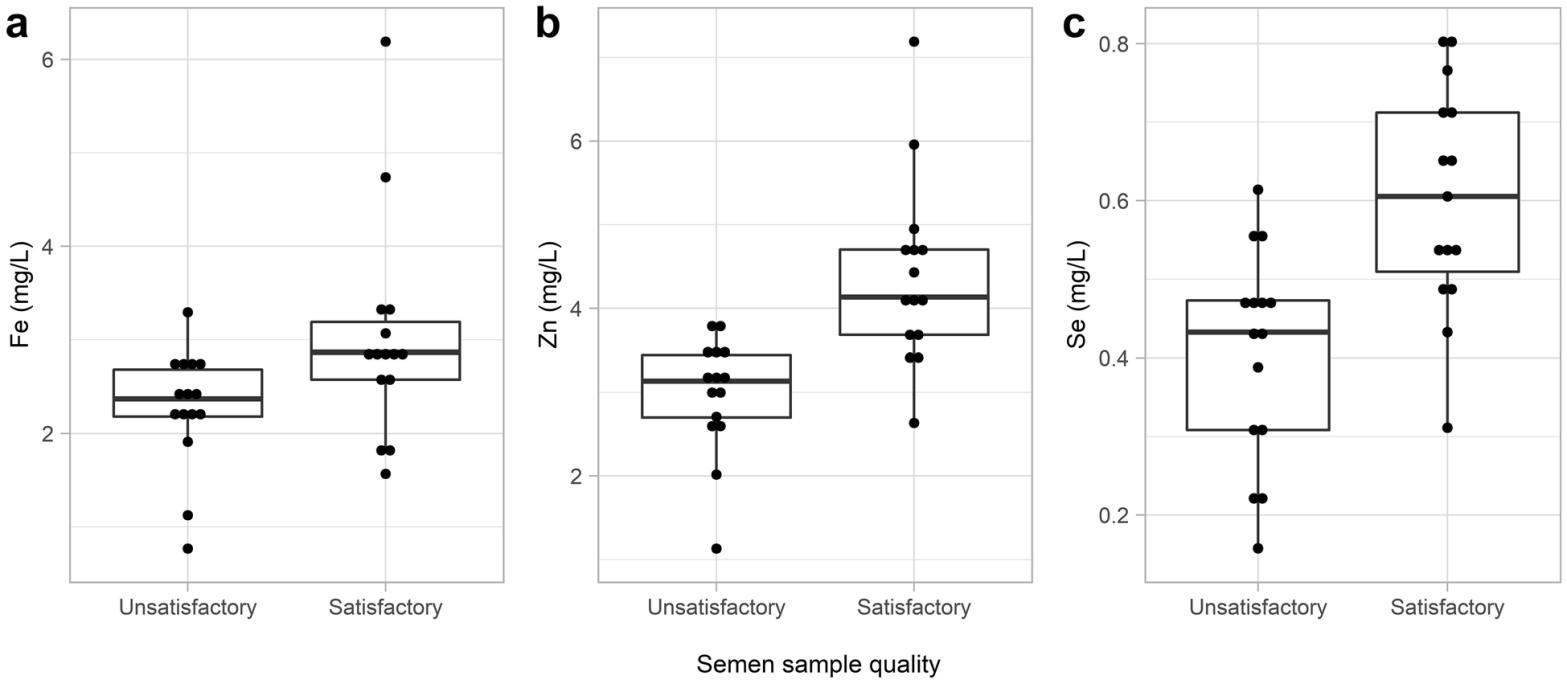
Concentration of Fe in fresh seminal plasma (**a**) and concentration of Zn (**b**) and Se (**c**) in blood serum in the satisfactory and unsatisfactory semen samples

The concentration of Fe in fresh seminal plasma was the only element in seminal plasma that was significantly higher in the SAT group than in the UNSAT group (P  =  0.0186; Fig. 1a). The concentrations of Zn and Se measured in blood serum were significantly higher in the SAT group than in the UNSAT group (P  =  0.0001 and P  =  0.0006, respectively; Fig. 1b, c).

### Correlation between element concentrations in seminal plasma and their concentrations in blood serum samples

A statistically significant correlation between the concentrations of elements in blood serum and in seminal plasma was found only for Cu and Na. Both correlations were statistically significant (P  <  0.001) and very strong, with Spearman’s ρ values of 0.8834 for Cu and 0.8220 for Na.

## Results of the multivariate regression model including elements

### Association between elements in fresh seminal plasma and the characteristics of fresh and frozen-thawed semen characteristics

The relationship between the concentrations of microelements in fresh seminal plasma and the characteristics of fresh and frozen-thawed semen was analyzed using a multivariate linear regression model. The results are summarized in Table 3. Fe was significantly associated with motility, progressive motility, the proportion of spermatozoa with acrosomal defects, and DNA fragmentation. Fe concentration was positively associated with motility and progressive motility, whereas it was negatively associated with acrosomal defects and DNA fragmentation. The values described above refer to fresh semen. In frozen-thawed semen, Fe was positively associated with viability and negatively associated with DNA fragmentation. Zn was negatively associated with acrosome defects. There were no statistically significant associations between semen quality characteristics and Se concentrations.

**Table 3:** Multivariate analysis for semen quality characteristics of fresh and frozen-thawed semen and elements Fe, Se and Zn measured in seminal plasma on the day of collection

Variables	Elements in seminal plasma (mg/L)					
	Fe		Se		Zn	
Semen characteristics (%)	β (95% CI)	P	β (95% CI)	P	β (95% CI)	P
Fresh semen						
Motility^a^	1,30,610 (38,101–2,23,118)	0.0075	−1,26,431 (−1,164,930 to 9,12,069)	0.8044	61,517 (−58,790 to 1,81,823)	0.3029
Progressive motility^a^	1,19,776 (56,976–1,82,575)	0.0006	−77,622 (−7,82,609 to 6,27,364)	0.8227	42,553 (−39,118–1,24,223)	0.2940
Acrosome defects^b^	−0.4749 (−0.8642 to −0.0855)	0.0188	2.3681 (−2.0026 to 6.7388)	0.2756	−2.0780 (−1.0140 to −0.0013)	0.0494
DNA fragmentation	−1.6851 (−3.1603 to −0.2099)	0.0268	−1.9652 (−18.5260 to 14.5956)	0.8092	−1.3558 (−3.2743 to 0.5627)	0.1583
Frozen-thawed semen						
Viability^a^	1,14,891 (24,909–2,04,872)	0.0143	−1,56,982 (−1,167,111 to 8,53,146)	0.7519	72,103 (−44,917 to 1,89,124)	0.2165
DNA fragmentation	−3.4984 (−5.7577 to −1.2391)	0.0038	−5.7146 (−31.0774 to 19.6481)	0.6471	−1.0197 (−3.9579 to 1.9185)	0.4820

^a^Cubic transformation

^b^Log transformation

### Association between elements measured in blood serum and the characteristics of fresh and frozen-thawed semen

The results of the multivariable regression analysis for the elements measured in blood serum are summarized in Table 4. Selenium was associated with almost all semen characteristics except acrosome defects in fresh and frozen-thawed semen and DNA fragmentation in fresh semen. The association with DNA fragmentation in frozen-thawed semen was negative, and all other quality characteristics were positively associated with Se concentration. Similarly, Zn was significantly associated with most semen quality characteristics. However, it was not associated with progressive motility and viability in fresh semen and normal tail membrane integrity, but it was negatively associated with the proportion of spermatozoa with acrosomal defects (P  =  0.0494). Fe measured in blood serum was significantly negatively associated only with DNA fragmentation in fresh semen.

**Table 4:** Multivariate analysis for semen quality characteristics of fresh and frozen-thawed semen and elements Fe, Se and Zn measured in blood serum on the day of collection

Variables	Elements in blood serum (mg/L)					
	Fe		Se		Zn	
Semen characteristics (%)	β (95% CI)	P	β (95% CI)	P	β (95% CI)	P
Fresh semen						
Motility^a^	80,738 (−39,156 to 2,00,632)	0.1781	8,02,538 (4,61,390–1,143,685)	0.0001	94,064 (39,659–1,48,469)	0.0015
Progressive motility^a^	82,652 (−20,842 to 1,86,147)	0.1127	6,49,692 (3,55,208–9,44,176)	0.0001	44,795 (−2169 to 91,758)	0.0607
Viability^a^	1,23,403 (−7048 to 2,53,853)	0.0627	7,82,244 (4,11,058–1,153,430)	0.0002	47,977 (−11,218 to 1,07,173)	0.1077
Normal morphology^a^	−5386 (−1,21,992 to 1,11,220)	0.9251	6,08,177 (2,76,385–9,39,969)	0.0009	1,03,614 (50,701–1,56,528)	0.0004
Acrosome defects^b^	−0.6213 (−1.3876 to 0.1451)	0.1076	−0.7251 (−2.9057 to 1.4555)	0.5003	−0.4135 (−0.7613 to −0.0658)	0.0216
Normal tail membrane integrity^a^	−85,817 (−2,25,635 to 54,001)	0.2183	5,05,314 (1,07,474–9,03,153)	0.0148	70,115 (6669–1,33,561)	0.0316
DNA fragmentation	−3.5201 (−6.4732 to −0.5670)	0.0213	−6.2227 (−14.6255 to 2.1802)	0.1400	−1.0848 (−2.4249 to 0.2552)	0.1081
Frozen-thawed semen						
Motility^a^	38,919 (−75,091 to 1,52,930)	0.4891	7,81,556 (4,57,150–1,105,962)	0.0000	71,343 (19,608–123,078)	0.0088
Progressive motility^a^	4328 (−65,223 to 73,878)	0.8992	2,33,317 (35,418–4,31,216)	0.0226	52,522 (20,962–84,083)	0.0021
Viability^a^	67,129 (−72,219 to 2,06,477)	0.3312	6,34,447 (2,37,944–1,030,950)	0.0029	95,076 (31,843–1,58,309)	0.0047
Normal morphology^a^	−59,182 (−1,73,925 to 55,561)	0.2988	5,48,513 (2,22,021–8,75,005)	0.0019	91,924 (39,856–1,43,992)	0.0012
Acrosome defects^b^	0.0237 (−0.4278 to 0.4752)	0.9149	−1.2739 (−2.5585 to 0.0107)	0.0518	−0.1664 (−0.3713 to 0.0385)	0.1070
Normal tail membrane integrity^a^	−19,935 (−1,43,509 to 1,03,638)	0.7428	8,14,528 (4,62,911–1,166,146)	0.0001	47,923 (−8152 to 1,03,998)	0.0907
DNA fragmentation	−1.5635 (−5.6921 to 2.5652)	0.4434	−13.9299 (−25.6775 to −2.1822)	0.0219	−2.4675 (−4.3410 to −0.5940)	0.0118

^a^Cubic transformation

^b^Log transformation

## Discussion

The results show an association between element levels in fresh bull seminal plasma and serum and sperm quality characteristics on the day of collection and after freezing and thawing. Zn and Se measured in serum were associated with almost all quality characteristics of fresh and frozen-thawed semen. In this study, differences were also found between bulls that met all criteria of semen characteristics (SAT) and bulls that did not meet at least one of the criteria of sperm characteristics (UNSAT) on the day of collection according to the concentration of Zn and Se in blood serum and Fe in seminal plasma. Data comparisons with the results of other studies are limited because few studies have measured microelements in the blood serum and seminal plasma of bulls to determine their influence on semen quality characteristics [4, 7, 9, 18–20]. Studies on dietary supplementation of microelements and their influence on semen quality or addition of some of the microelements directly into the semen extenders were of highest interest to the researchers [21–26]. The results of dietary supplementation with Se or parenteral administration of Se had contradictory results. In some studies, no difference in semen quality was found after supplementation [27, 28], which may be related to the fact that mineral supplements commonly fed to livestock are commercially available in inorganic form. It is now known that trace minerals are present in the body almost entirely as organic complexes or chelates [29]. Organic trace mineral element sources may be more bioavailable, possibly by reducing negative interactions that could affect their efficiency of conversion to a physiologically active form [19, 30]. In a study in which organic Se was supplemented in food and feed to buffalo bulls for three months, the semen quality characteristics (ejaculate volume, sperm motility, concentration, and morphology) were significantly improved [19]. In addition, researchers have found that the addition of Se directly into semen extenders improved postthaw semen characteristics [7, 31].

Selenium measured in fresh seminal plasma was associated with the proportion of spermatozoa with acrosome defects in fresh semen, consistent with the results of increased sperm acrosome integrity and number of sperm bound to the zona pellucida when Se was added to the fertilization medium [20].

In our study, Se measured in blood serum was associated with almost all the characteristics in fresh semen and with all the characteristics of frozen and thawed semen and therefore could serve as a good predictor of semen quality after freezing and thawing. Since Se is involved in normal spermatogenesis and represents an essential component of a number of selenoproteins (e.g., glutathione peroxidase 4-GPX4), its concentration in blood is crucial for the normal development of spermatozoa. GPX4 activity is highest during male germ cell development [32]. When Se concentrations are low during the final stages of spermatogenesis, GPX4 activity is decreased, leading to higher concentrations of active peroxides that can cause oxidative injury that can accumulate and lead to delayed viability impairment [33]. Moreover, Parillo et al. [34] reported that strong expression signals of GPX4 protein were observed in the apical region of seminiferous tubules in bulls. These dynamic signals at different stages of sperm maturation suggest that GPX4 is essential for optimal bovine sperm development and function and that Se deficiency may lead to impaired spermatogenesis [34]. However, this last phase of spermatogenesis in bulls lasts 17 days; therefore, the concentration of microelements measured in the blood serum on the day of sperm collection could not truly be correlated with spermatogenesis itself. Since all bulls were fed the same diet, we believe that the same observations would be found even if the blood serum had been collected before sperm collection; however, this assumption should be tested in a future study. The statistically significant difference in Se concentration in blood serum between bulls in the SAT and UNSAT groups in our study could also be explained by the strong expression of GPX4 in epididymal fluid, prostatic fluid and seminal vesicular fluid in fertile bulls [34].

Additionally, Zn measured in blood serum was associated with almost all quality characteristics of fresh semen and all quality characteristics of frozen-thawed semen characteristics in our study. Moreover, when we established significant differences among trace elements between the SAT and UNSAT groups, Zn measured in blood serum was significantly higher in the SAT group. Similar to our results, Zn deficiency also caused reduced male fertility in the study by Kerns et al. [35]. Zn is involved in epididymal sperm maturation and capacitation [35], and its higher concentrations in seminal plasma positively correlate with progressive motility [36]. Additionally, Zn measured in blood serum was associated with almost all fresh semen quality characteristics and all thawed semen quality characteristics in our study. Moreover, when we found a significant difference between trace elements between the SAT and UNSAT groups, Zn measured in blood serum was significantly higher in the SAT group. Similar to our results, Zn deficiency also caused reduced male fertility in the study by Kerns et al. [35]. Since Zn is involved in epididymal sperm maturation and capacitation [35] and its higher concentrations in seminal plasma positively correlate with progressive motility [36], the association between blood serum Zn concentration and semen quality is consistent with better semen characteristics in our study, in which a higher proportion of morphologically normal sperm (in fresh and freeze-thawed semen), a higher proportion of sperm with intact tail membrane integrity, and progressively motile sperm (in freeze-thawed semen) were observed. Our results suggest that Zn measured in blood serum may be a good predictor of sperm quality characteristics after freezing and thawing.

In the male reproductive system, Fe can play either a positive or negative role depending on the Fe concentration. Increased Fe concentration in rat testes was associated with oxidative damage to lipids, proteins, and DNA [37]. On the other hand, Fe deficiency reduced the activity of Fe-containing and Fe-dependent enzymes [15]. In our study, Fe measured in blood serum and seminal plasma was positively associated with better maintenance of semen quality characteristics. More specifically, Fe in blood serum was positively associated with viability and negatively associated with the proportion of spermatozoa with acrosome defects and DNA fragmentation. Fe measured in seminal plasma was positively associated with motility, progressive motility, viability, morphologically normal spermatozoa, and spermatozoa with normal tail membrane integrity. Similarly, a negative association with acrosome defects was observed. Higher motility and viability are consistent with the study of Eghbali et al. [38], where the Fe concentration in seminal plasma was associated with the motility and viability of the spermatozoa after ejaculation. In our previous study investigating microelements in the seminal plasma of boars, a higher Fe content was associated with a higher percentage of live spermatozoa and spermatozoa with normal morphology after 3 days of storage [16]. In bulls, the addition of FeCl_2_ to diluted semen samples at concentrations above 50 μmol/L was shown to result in a significant decrease in sperm motility and mitochondrial activity. However, concentrations below 10 μmol/L FeCl_2_ stimulated spermatozoa activity, as evidenced by significant preservation of motility and viability characteristics in dilute bull spermatozoa [9]. In our study, when the mean Fe concentration in bull seminal plasma was 2.65 mg/L, higher Fe levels correlated with better semen quality. From multivariate analysis, Fe also affected the tail membrane and DNA integrity. Spermatozoa are under increased oxidative stress during storage [39]; therefore, low Fe concentrations may result in lower activity of Fe-dependent enzymes such as catalase [15]. These factors could cause an increase in lipid peroxidation in bull spermatozoa and lead to reduced viability.

In our study, positive correlations between element concentrations in seminal plasma and serum were significant only for Cu, Fe, and Na levels. A positive correlation for Fe was also found in the study of Dhami et al. [40]; however, a negative association between blood and semen elements was also found for Cu and Zn levels in their study. The exact mechanism by which elements are transferred from circulating blood to seminal plasma is unclear [41]. In agreement with other studies in humans, in which the relationship between elements in blood and in semen plasma has not been confirmed [42, 43], no significant correlations were found between Se concentration in blood and Se concentration in seminal plasma in a study conducted in stallions [44].

A recent study showed that spermatozoa from individual bulls differ in their ability to survive cryopreservation [45]. In our study, bulls that met all the requirements for semen characteristics had higher Zn and Se concentrations in blood serum and higher Fe concentrations in seminal plasma compared to bulls that did not meet at least one of the requirements for good semen characteristics. Therefore, the concentrations of Zn, Se and Fe in the blood serum and Zn Fe in the seminal plasma of bulls can serve as freezability biomarkers.

## Conclusions

Microelements in bull seminal plasma have been associated with bull sperm quality and could be used as an additional tool to predict semen quality after thawing. Due to the significant correlations between Fe, Zn and Se concentrations in blood serum and in fresh seminal plasma and semen characteristics after thawing, measurements of the elements may be useful to discriminate between high- and low-freezability semen samples.

## Data Availability

The datasets used and/or analyzed during the current study are available from the corresponding author on reasonable request.
